# Detection of Antibodies against *Mycobacterium bovis* in Oral Fluid from Eurasian Wild Boar

**DOI:** 10.3390/pathogens9040242

**Published:** 2020-03-25

**Authors:** Jose A. Barasona, Sandra Barroso-Arévalo, Belén Rivera, Christian Gortázar, Jose M. Sánchez-Vizcaíno

**Affiliations:** 1 VISAVET Health Surveillance Centre and Animal Health Department, Faculty of Veterinary, Complutense University of Madrid, 28040 Madrid, Spain; 2 SaBio. Instituto de Investigación en Recursos Cinegéticos (IREC; CSIC-UCLM), Ronda de Toledo s/n, 13003 Ciudad Real, Spain

**Keywords:** animal tuberculosis, antibody detection, ELISA test, disease surveillance, oral fluid, *Sus scrofa*

## Abstract

The presence of *Mycobacterium bovis* and other members of the *Mycobacterium tuberculosis* complex (MTC) is a main concern in wildlife populations such as the Eurasian wild boar (*Sus scrofa*). Tests detecting antibodies against the MTC are valuable for tuberculosis (TB) monitoring and control and particularly useful in suids. The development of accurate, efficient, and non-invasive new tools to detect exposure to MTC would be highly beneficial for improving disease surveillance. This study aimed to determine if antibodies against MTC could be detected in oral fluid (OF) samples by a new ELISA test (IgG detection) from naturally TB-infected wild boar. For this, individual, paired serum and OF samples were collected from 148 live wild boar in two TB-status areas from Spain and quantitatively used to validate the new ELISA test. Antibodies against MTC were widely detected in OF samples, for which a significant positive correlation (*r* = 0.83) was found with the validated serology test. OF ELISA sensitivity and specificity were 67.3% and 100%, respectively. The results of this work suggest that OF samples have the potential to be used for MTC diagnosis as a further step in TB surveillance and control in suid populations. Based on our results, further research is warranted and could be performed using non-invasive new tools directly in field conditions to detect exposure to MTC.

## 1. Introduction

Tuberculosis (TB) is caused by infection with *Mycobacterium bovis* and other close members of the *Mycobacterium tuberculosis* complex (MTC) [1]. TB, an infectious and contagious disease, is nowadays one of the most widespread examples of a shared infection that is prevalent in both wildlife and livestock [2]. *M. bovis* is transmitted among wildlife by different routes of infection, including direct and indirect paths [3,4]. Although TB has been reduced and even eliminated in livestock in many countries, wild species can act as a reservoir of infection, contributing to its maintenance. The role of wild hosts in TB epidemiology can differ among areas [5]. Within wildlife species in continental Europe, the Eurasian wild boar (*Sus scrofa*) represents one of the greatest risks for the spread of this disease [6] and plays a crucial role in the maintenance of *M. bovis* in the Mediterranean ecosystems of the Iberian Peninsula [7,8]. Wild boar not only are able to maintain the circulation of *M. bovis* in the absence of livestock [5] but also are the main species transmitting this pathogen in multi-host communities, at least in managed scenarios [9].

In recent years, wild boar populations have alarmingly increased throughout the European continent [10,11,12]. This implies not only a larger number of hosts available for the transmission of *M. bovis* but also a higher interaction between hosts [10,13]. Because of all these reasons, in addition to their behavior, feeding habits, ability to cross barriers and interact with other wildlife and livestock, and susceptibility to mycobacterial infections, wild boar are used as sentinels of TB in official surveillance programs [14,15].

Thus, monitoring and mitigating the transmission of *M. bovis* among wildlife should become a priority for ensuring TB eradication in livestock [16]. The early detection of the disease in the source of the infection and the sanitary surveillance of control actions in wild boar populations (culling, vaccination, biosafety, etc.) [17,18] may help to limit disease transmission to livestock. Therefore, the development of diagnostic methods and new tools to detect exposure of wild boar to mycobacteria would be valuable for improving disease surveillance and wildlife management. Although the gold standard confirmation for *M. bovis* infection is microbiological culture following by identification of the agent [19], serological methods are increasingly used in wildlife and livestock screening because they are rapid, simple, relatively low-cost, and also suitable for retrospective studies [20]. At present, reliable antibody assays are available for the diagnosis of MTC infection in wild boar [17]. Concretely, one of the most used techniques is the Enzyme-Linked Immunosorbent Assay (ELISA), which is often recommended for wild boar TB monitoring [14,17]. Typically, these kinds of test are performed on blood serum samples. However, the cost of collecting and testing enough samples is high, making the implementation of active surveillance difficult. Besides, conventional in vivo sampling procedures involve the risk of mortality, since the capture and either physical or chemical immobilization induce stress in the animals [21,22].

Among possible alternative samples, oral fluid (OF) seems to be a good option, since it has already been adapted for the detection of other infections in wild and domestic swine. Oral fluids have been previously used to early detect foot-and-mouth disease and classical swine fever virus infection in wild boar using a rope-in-a-bait procedure [23,24]. Besides, recent studies demonstrated the value of OF for the antibody detection of a variety of swine pathogens including porcine reproductive and respiratory syndrome (PRRS) [25], porcine circovirus type 2 [26], and African Swine Fever virus (ASF) [27] in pigs, among others. In contrast to serum samples, collecting OF samples from wild boar using ropes is an easier and less stressful method (welfare-friendly), based on their natural chewing and investigatory behavior [28,29]. Additionally, this procedure is more sensitive than individual sampling for detecting infections in populations, leading to a higher probability of detection of certain pathogens with a smaller number of samples [30].

Therefore, we aimed to determine if antibodies against MTC could be detected in OF samples from free-range wild boar naturally infected, as the first step in evaluating their potential use for TB surveillance and control in wild boar populations. In addition, individual, paired serum and OF samples were used to validate the new test.

## 2. Results

### 2.1. Descriptive Analysis

**Figure 1:**
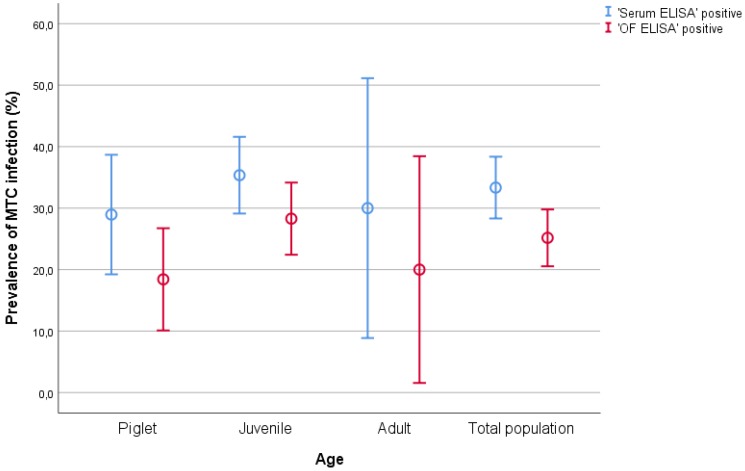
Prevalence of *Mycobacterium tuberculosis* complex (MTC) infection according to age in serum (Serum ELISA, in blue) and oral fluid (OF ELISA, in red) samples, determined by ELISA. Error bars denote the standard error values.

Table 1 shows the age distribution of the population screened and the proportion of positive and negative results of OF and serum samples for each age (Figure 1). All positive samples came from the TB-endemic site (Sevilla), while in the TB-free site (Albacete) the apparent prevalence was 0%. No animals showed any symptoms or clinical signs compatible with TB infection.

### 2.2. OF ELISA Validation

**Figure 2:**
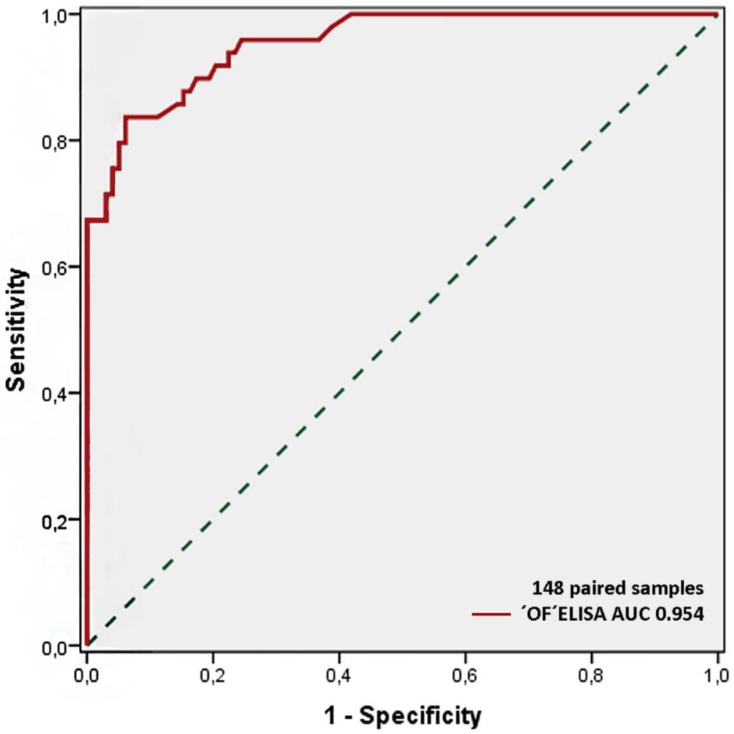
The receiver operating characteristic (ROC) curve drawn to obtain the optimal cut-off value for OF ELISA in detecting MTC infection.

The area under the curve (AUC), indicating accuracy, was determined for the mean of the optical density (ODs) duplicates and was equal to 0.954 (Figure 2). The best cut-off point, determined by the receiver operating characteristic (ROC) analysis, was 0.211 for the OD values. Based on this cut-off value, OF ELISA samples’ sensitivity and specificity were 67.3% and 100%, respectively. The test reported a 100% positive predictive value (PPV), an 89% negative predictive value (NPV), and a 95.4% level of accuracy (95% CI: 0.963–1.000). Both tests had a kappa agreement of 0.804. ‘OF ELISA’ ODs and ‘Serum ELISA’ ODs showed a positive correlation (*r* = 0.832, *p* = < 0.001) (Table 2).

## 3. Discussion

This study shows the ability of the first test for detecting antibodies against MTC antigens in OF from animals. The results confirm that OF samples can be useful for TB diagnosis in naturally infected free-ranging wild boar. The current epidemiological situation in countries where TB is still prevalent indicates that wildlife is a key factor for the expansion and maintenance of the disease [5]. With this scenario, continuous improvements in sampling techniques and diagnostic procedures in wildlife are essential tools for disease control or, at least, disease surveillance. In particular, wild boar act as a reservoir and source of the infection [8,31], favoring the propagation to livestock and, therefore, to humans [32]. In contrast to other species, the antibody response to MTC infection in suid species is easily detected and maintained over time [14,33]. Thus, the development of a diagnostic test easy to implement in the field, with low-cost specimen collection and high specificity, may contribute to a better understanding of the infection dynamics [34]. Herein, a new ELISA based on a non-invasive sampling (pathogen sampling of wild animals by baits; pSWAB) technique for OF collection from wild boar was developed and tested for its suitability to timely detect MTC infection in field conditions.

Although bacterial culture is considered the gold standard technique for the diagnosis of mycobacterial infections in wild boar [35], it requires access to the individual, which is not always feasible as a surveillance measure. Additionally, its sensitivity depends on the number and selection of tissues processed [36] and on sample quality [37]. Thus, bacterial culture and other post-mortem methodologies were not considered for this study, where no animals were culled. On the other hand, antemortem TB testing of wild boar has previously been attempted using serology, tuberculin skin test, and gamma interferon assay. Both serology and gamma interferon tests require capturing and managing the animals to extract fresh blood samples, an equipped laboratory environment, as well as skilled personnel [14,35]. The tuberculin skin testing requires handling the hazardous wild boar repeatedly and has poor specificity rates [38], while antibody detection is faster, cheaper, and simpler, and more suitable for live animals [39]. Current TB antibody tests are based on the use of serum as a sample, which requires operators and a larger amount of resources. However, OF has become a viable alternative to serum because it is easily collected and provides equivalent information [40,41], as supported by the preliminary results from this pilot study, which showed the potential of this new approach for future research.

Our results showed a high correlation between the serum sample test and the OF sample test (OF ELISA), indicating that typical serum ELISA may be complemented by the new OF ELISA, with the advantages that this entails: (1) pSWABs OF sampling does not require the direct involvement of operators. Instead, ropes can be placed in the area frequented by wild boar, taking advantage of their natural chewing behavior. The ropes have been found to be attractive to wild boar, especially in areas with resources or supplementary food, facilitating OF collection [42]; (2) samples are easier to collect— therefore the metod is less stressful (welfare-friendly)—transport, and store; therefore, (3) this technique can be directly applied in field conditions, facilitating the implementation of surveillance and eradication measures [43].

This study has limitations for the lack of confirmatory results based on the gold standard. The main objective of this study was to develop a new antibody-based test suitable for live animals, without performing a necropsy. Therefore, we could not perform a bacterial culture of target tissues. The “Serum ELISA” used in our pilot study as a reference test has previously shown a sensitivity ranging from 86.4% (in natural wild boar infections) to 94.4% (in experimental wild boar infections, [44]), and a specificity of 100% [45]. Diagnostic accuracy may be underestimated when a reference standard with reduced sensitivity is used. Although the reported accuracy will always be lower than the true accuracy, the sensitivity will increase towards its original value as prevalence increases [46]. In addition, considering the current TB scenario in the Iberian Peninsula (high proportion of TB-positive animals, [47]), this loss of sensitivity can be assumed since diagnosis is normally performed at the population level, providing NPV and PPV are adequate, which was the case. The cutoff was selected to maximize specificity and ensure good sensitivity. OF ELISA sensitivity was 67.3% regarding the reference test, while specificity was 100%. The lower sensitivity achieved in OF sample testing was probably due to the lower antibody concentration in OF compared to serum. For instance, it has been previously described that the IgG concentrations in OF are ca. 800 times lower than in serum [48]. However, for routine surveillance in field conditions, OF may be preferable to serum, because rope samples provide improved detection over single-animal testing [23]. PPV for OF ELISA was excellent (100%), while NPV was also high but lower than PPV (89%), as may be expected for a disease with such a high prevalence [49]. On the other hand, the number of false negatives (FN) from OF ELISA was relatively high (10.81%). Consequently, the lower sensitivity of OF ELISA has to be taken into account when selecting a diagnosis test. For instance, MTC antibody detection from oral fluid may not be accurate enough for individualized studies where high precision is required. Instead, it may be appropriate in other kinds of studies where practical methods are needed. This fact is particularly important in the case of collective diagnosis, since simplicity may play a key role during the sampling. Despite the generalized low sensitivity of antibody detection in wild boar piglets [17], this test has also maintained a high correlation with the results obtained by serology in this age class. These values indicate that positive results are very powerful clues for the detection of MTC antibodies, but negative serological tests cannot exclude MTC presence. Because of the dynamics of the infection, both values seem to be adequate for a new diagnostic test whose main target is the wild boar population. Another issue of concern is the low number of adults available. As infected wild boar are commonly able to survive despite this chronic infection, the reliability of the test to detect infected adult animals is essential. Therefore, further validation in a larger sample and especially with more adult wild boar is required.

Since none of the animals showed any symptoms, as is common in wild boar, we were not able to study the relationship between clinical findings and the ELISA results. However, wild boar frequently act as carriers of the disease, and, within this scenario, post-mortem evaluation may be the only way to establish this relationship.

Overall, this new approach in detecting MTC infection has the potential to be a reliable and efficient complement as a screening diagnosis by using a non-invasive collection method (pSWAB) in wild boar populations. Although the results obtained from this pilot study require further validation, alternative strategies for effective population-level monitoring of TB such as the pSWAB-methodology could assist in better targeting field interventions in settings where sampling of individual animals is challenging, despite their reduced sensitivity compared to blood sampling. Hence, the applicability of this new methodology is high, especially in areas where the TB prevalence is low or unknown. This finding has important implications for developing and implementing surveillance and control programs for TB in wildlife.

## 4. Material and Methods

### 4.1. Animal Sampling

We conducted the non-invasive sample collection in two separate areas of Spain with different wildlife management and TB status. On one side, an intensively managed private hunting estate (TB-endemic site) in Sevilla province, southern Spain, was sampled during 2017. Wild boar TB prevalence in this site ranged between 34% and 68% from 2011 to 2015 [17,50]. In this intensively managed estate (for hunting), wild boar piglets and yearlings (hereafter, juvenile wild boar) are captured from spring to summer and maintained in enclosures for about 3 months until hunting season opening. Juvenile wild boars are released back into the wild after the first hunting event. This procedure is expected to reduce juvenile mortality during the drier summer months and the first hunting event [50]. A total of 133 wild boar (70 males and 63 females) were managed and sampled for this study in the TB-endemic site.

The second site is a nature reserve (TB-free site) located on the Ruidera Natural Park (Guadiana Valley), Albacete province, eastern Spain, sampled during 2017. The study site has moderate densities of wild boar, but for now, the TB prevalence in wild boar and livestock is very low in Guadiana Valley [7,51,52]. The capture operations of wild boar in this site, by contrast, are sporadic and carried out to maintain the density of wild boar under permissible critical thresholds within the protected area, since it is a wetland with high ecological value due to its population of waterfowl [53]. In this way, wild boars are captured and, after sanitary monitoring, they are released back into the wild in managed hunting estates. A total of 15 wild boar (10 males and 5 females) were captured and sampled for this TB-free site.

### 4.2. Study Design

The study was performed in accordance with EC Directive 86/609/EEC and followed the recommendations provided in 2007/526/EC regarding the accommodation and care of animals used for experimental and other scientific purposes (Eur-lex.europa.eu). The study was conducted under a research license (828493/2013) issued by the University of Castilla-La Mancha and was developed by veterinary scientists with B and C animal experimentation categories. No animals were culled for the study.

Overall, a panel of individual, paired OF and serum samples from unknown TB-status wild boar was created by collecting samples from 148 animals. Sources and sampling periods are summarized in Table 3.

### 4.3. Serum Sample Collection and Testing (Serum ELISA)

Blood samples (n = 148) were collected from the orbital venous sinus [54] by venipuncture, using evacuated tubes without additives (Vacuette, Deutscher SAS, Brumath, France). Serum was purified by centrifugation for 10 min at 3500× *g* and then stored at −20 °C until analysis.

Serum samples were tested for anti-PPD immunoglobulin antibodies using a commercial ELISA, using bovine tuberculin purified protein derivative (bPPD) as the antigen and protein G horseradish peroxidase as a conjugate and applying a previously described protocol [45]. Sample results were expressed as an ELISA percentage (E%) that was calculated using the following formula:

[sample E% = (mean sample OD/2 × mean negative control OD) × 100]

Cut-off values were defined as the ratio of the mean sample OD to the sum ODs of the negative controls. The cut-off with the best specificity was chosen. Serum samples with an E% value greater than 100 were considered positive.

### 4.4. Oral Fluid Sample Collection and Testing (OF ELISA)

Oral fluid samples were obtained from the same animals as those used for serum collection, using a rope-in-a-bait sampling technique (referred to as pSWAB). All samples were collected individually, ensuring that each animal actively bit the rope for four minutes, enough time to obtain a similar amount of oral fluid from all animals. A cotton rope of 12 mm of thickness and 15 cm of lenght (RopeServices UK, Houghton Le Spring, UK) with corn dust was chewed by the wild boar during the management procedures. After being chewed, each rope was manually stored in watertight bags at 4 °C and sent to the laboratory, where it was processed in a maximum of 2 days after collection. pSWABS samples were previously processed to extract OF. Briefly, ropes from pSWABS were introduced in a 20 mL syringe within a 50 mL tube. After the addition of 5 mL of phosphate-buffered saline (PBS), the tubes were centrifuged at 4000 rpm for 10 min, and the supernatant was used for the diagnostic test.

To develop the ELISA of OF samples (OF ELISA), plates were coated with bPPD tuberculin at 50 mg/mL (100 μL of final volume per well) and incubated overnight at 4 °C. Following one wash with PBS-Tween-20 (PBS-T; 0.05%), the plates were blocked with 280 μL of tris-buffered saline with 0.05% Tween-20 (TBS-T). A skim milk powder solution (5% concentration) was added, and the plates were incubated for 1 h and 30 min at 4 °C. The buffer was removed, and the plates were allowed to dry at room temperature for 1 h. Then, the OF samples were diluted 1:8 with sample diluent and TBS-T and incubated for 1 h at 37 °C. The plates were washed three times with PBS-T, and goat anti-swine horseradish peroxidase (HRP)-conjugated IgG (Jackson ImmunoResearch Laboratories, West Grove, PA, USA) at a 1/15.000 dilution was added to each well. Then, the plates were incubated for 30 min at 37 °C and subsequently washed with PBS-T three times. Finally, color was developed by adding 100 μL per well of Sureblue Reserve TMB Microwell Peroxidase Substrate (TMB; KPL, Gaithersburg, MD, USA). The plates were protected from the light and incubated at room temperature for 15 min. The reaction was stopped ba adding 50 μL of H2SO4 (3 M) to each well. Absorbance at 450 nm was determined using an Anthos 2001 plate reader (Labtec, Salzburg, Austria). ELISA results for IgG were also expressed by using the formula cited above: [sample E% = (mean sample OD/2 × mean negative control OD) × 100].

### 4.5. Statistical Analysis

The data were analyzed using the commercial statistical software SPSS 22 (IBM, Chicago, USA). Correlation analysis between ELISA optical densities (OD) in OF and serum samples was performed using Pearson’s ranked coefficient test. Cohen’s kappa coefficient (κ) was used to calculate the degree of agreement of the OF test (OF ELISA with the standard Serum ELISA). ROC analyses were performed to estimate the AUC by using serology data from known uninfected and MTC-infected wild boar and to determine cut-off values [55]. The cut-off values were calculated according to the Youden’s index [56]. Sensitivity and specificity for the new OF ELISA test were calculated against the standard Serum ELISA results. Sensitivity was calculated as the proportion of test-positive samples over the total number of samples from seropositive animals. Specificity was calculated as the proportion of test-negative samples over the number of seronegative animals. Positive predictive value (percentage of animals with a positive test who actually have the disease, PPV), and negative predictive value (percentage of animals with a negative test who do not have the disease, NPV) were calculated according to Parikh et al. [49]. Confidence intervals for proportions were calculated using the modified Wald method in Graph Pad (GraphPad Software, Inc., La Jolla, CA, USA). Analyses were considered significant at *p* < 0.05.

## Figures and Tables

**Table 1:** Distribution of positive animals and apparent prevalence for serum and oral fluid tests according to animals’ age. OF: oral fluid.

Age	Serum Test Positive (Prevalence)	OF Test Positive (Prevalence)	Total Sampled
**Piglet**	11 (28.2%)	7 (17.9%)	39
**Juvenile**	35 (35.3%)	28 (28.8%)	99
**Adult**	3 (30%)	2 (20%)	10
**Total population**	49 (33.1%)	37 (25%)	148

**Table 2:** Two-by-two table used to determine the sensitivity and specificity of the oral fluid ELISA test. TP: true positives; FP: false positives; FN: false negatives; TN: true negatives.

Sample Type	Reference Serum Positive	Reference Serum Negative	Total Test Results
**Oral fluid ELISA test positive**	37 (TP)	0 (FP)	37 (total test positives)
**Oral fluid ELISA test negative**	12 (FN)	99 (TN)	111 (total test negatives)
**Total samples analyzed**	49	99	148 (total population)

**Table 3:** Description of the wild boar (*Sus scrofa*) samples used in this study. TB: tuberculosis.

Area	Sampling Period	Type of Sample	No. of Animals	Total No. of Animals
**TB-endemic site (Sevilla)**	February 2017	Serum	24	133
		Oral fluid		
	June 2017	Serum	66	
		Oral fluid		
	November 2017	Serum	43	
		Oral fluid		
**TB-free site** **(Guadiana Valley)**	June 2017	Serum Oral fluid	15	15
